# The Impact of Chlorinated Water and Sun Exposure on the Durability and Performance of Swimwear Materials

**DOI:** 10.3390/polym16213050

**Published:** 2024-10-30

**Authors:** Vesna Marija Potočić Matković, Ivana Salopek Čubrić, Katarina Krstović

**Affiliations:** Department of Textile Design and Management, University of Zagreb Faculty of Textile Technology, 10000 Zagreb, Croatia; ivana.salopek@ttf.unizg.hr (I.S.Č.); katarina.krstovic@ttf.unizg.hr (K.K.)

**Keywords:** material, swimwear, polyamide, polyester, yarn, aging, tensile properties, moisture management

## Abstract

Understanding the factors that affect how materials age is essential for creating a durable product with long-lasting properties. It is also important to prioritize defining aging parameters that reflect the real-world conditions the materials will encounter. For this study, a range of swimwear materials were selected consisting of a blend of polymer (polyamide/polyester) and elastane in varying ratios. In order to simulate aging conditions, materials were immersed in chlorinated outdoor pool water during the summer season, either in shade or the sun, for 200 and 300 h. The materials were tested for mass per unit area, thickness, tensile properties, and moisture management. A slight mass per unit area increase was observed, rising from 1.0% after 200 h of chlorine and sunlight exposure to 3.7% after 300 h. Thickness increased by 1.7% after 200 h and 3.2% after 300 h of chlorine exposure, with no significant effect of sunlight. Breaking force dropped by 12.4% after 200 h in chlorine and 8.2% in chlorine and sunlight, becoming more pronounced after 300 h (65.7% in chlorine and 65.1% in chlorine and sunlight). The overall moisture management capability declined from 0.4888 to 0.3457 after 200 h in chlorine and 0.3393 with sunlight, dropping further after 300 h to 0.3838 and 0.3253, respectively.

## 1. Introduction

Understanding the parameters that influence the response of materials to aging is critical to the development of a functional product whose properties should be maintained for as long as possible. Appropriate emphasis should be placed on defining aging parameters that take into account the conditions under which the materials must function. Among the external parameters that influence aging, some of the most important are radiation, temperature, humidity, oxygen, precipitation and condensed moisture, particulate and gaseous contaminants, and stress [1]. These parameters can build up a wide range of conditions under which material must operate. Therefore, it is important to understand the range and significance of each aging parameter in order to conduct an investigation. It is also important to understand why these parameters need to be considered, what influence they have, how their various combinations can determine the outcome, and what needs to be done to limit the damage.

The aging of materials under defined conditions has been investigated in various areas of scientific interest [2,3,4,5,6,7,8]. Scientists pointed out that thermal degradation under wet conditions is a significant aging mechanism in polyamide 6,6 (PA 6,6). Therefore, [9] investigated thermal degradation through reactive force field molecular dynamics (MD) and hyperdynamics simulations at temperatures from 1000 to 2000 K. The goal of the study was to examine the impact of water on the activation energy and pre-exponential factor of amide bond cleavage, aligning with experimental data and offering a predictive tool for long-term degradation analysis. The focus of the study conducted by Jun et al. [10] was the degradation of full aromatic PA membrane by sulfuric acid and hydrogen halides. The results of testing indicated that the exposure to sulfuric acid did not result in notable changes in the membrane but the water flux trough the PA membrane was severely decreased due to its halogenation. Researchers [11] also simulated the aging of polyethylene terephthalate material in water at temperatures above 110 °C. According to the results, a 5-month exposure leads to embrittlement with chain scission during hydrolysis if the molar mass of the material falls below 17 kg/mol. Recent findings on polymer aging in water confirm chain scission, and they show an increase in crystallinity due to aging [12]. In another study, a model for predicting the crystallinity changes has also been proposed [13]. Experiments with polymer materials aged only by solar radiation [14] showed a decrease in tensile strength while the enthalpy of fusion increased. It was also shown that aging led to a slight increase in the peak of the hydroxyl group at 3400 cm^−1^. Nanoscale analysis of the photochemical degradation of polyester fibers due to aging was also carried out. A peak was observed in the Raman spectrum and the factors of temperature, humidity, and radiation were taken into account. The results indicate that the degradation of a polymer is not uniform over the entire surface of the fiber when coupled with weathering [15].

In addition to the change in polymer properties, the accumulation of microplastics is another problem related to the aging of polymers that has been increasingly addressed recently. More specifically, the problem of the presence of plastics in the oceans, from the surface to sea floor sediment, has been investigated in recent studies [16,17,18,19,20,21,22,23,24,25,26]. Polyester, while not harmful to health and the environment per se, is a cause for concern due to its large volume in waste and low biodegradability, leading to the release of microplastics into the environment. Regarding polyester and the release of microplastics, researchers suggest that the main goal is to produce polyester with ideal structural properties and to produce as much that can be recycled as possible [27].

Polyester, polyamide 6, and polyamide 6.6 are frequently used in all textile products as they ensure long dimensional stability in contact with water, have excellent wear and abrasion resistance, and have a short drying time. For products worn directly on the skin, these polymers are often blended with elastane in various proportions to ensure comfort, optimum fit, and flexibility. The number of studies investigating the aging of materials is still quite limited. So far, researchers have shown that the aging of polymers under the influence of swimming pool conditions can lead to the degradation of the polymers in the crystalline phase, and ultimately to the breakage of the polymer chains [28]. As for elastane as a component of the material structure, the tests have confirmed the increased heat sensitivity at higher temperatures compared to other polymers [29,30].

Nevertheless, the question arises as to how the properties of polymeric materials change with aging, and whether aging under certain conditions drastically affects the properties required for optimal athlete performance. Our previous studies on the aging properties of polymeric materials used for sports purposes have been conducted in two directions—on the aging of materials under conditions such as those found in football and swimming. Regarding the effects of polymers used for football sportswear, the results showed a significant impairment of the polymer material’s ability to absorb moisture [31], and a decrease in force at the break for standard polyester fabrics with elastane of up to 26% [32]. Such changes can have a negative impact on the material in its normal use. The aging of PES and PA materials in the swimming pool showed even greater changes in the material properties. For example, the decrease in the force at the break of polyamide materials due to aging was up to 40%, which indicates a significantly reduced durability [33,34]. Another study focusing on the aging of knitted fabrics [35] indicated a notable reduction in tensile properties of PET knitwear, though the structural parameters, such as horizontal and vertical density, remained largely unchanged. As far as the water vapor resistance is concerned, it was showed that the water vapor resistance of PA- and PET-coated fabrics decreased after being exposed to outdoor conditions. The average drop in water vapor resistance was 11.4% after summer exposure and 16.7% after winter exposure [36]. Additionally, in another study, the heat resistance of PES-coated PA and PET fabrics diminished by 13% and 25% after three months of summer and winter weathering, respectively. SEM analysis revealed deterioration in the PES coating, but the PA and PET fabric substrates were unaffected [37].

This literature review has shown that there is a lack of research on the specific conditions of aging of polymers used in the manufacture of sportswear. In today’s world, where sport and physical activity play an important role in human well-being, the importance of such research cannot be underestimated. The purpose of research covering aging tests is to simulate the conditions that sportswear will encounter over time in order to assess the changes in material properties that directly affect durability, comfort, and performance of the material. Therefore, this study presents the results of an investigation into the aging of polymer materials used in the manufacture of swimwear. The value of researching swimwear materials through aging tests lies at the intersection of customer satisfaction, material innovation, sustainability, and market competitiveness. By understanding how fabrics age, manufacturers can enhance product quality by designing materials that can withstand harsh conditions typical for swimwear, and they can reduce environmental impacts by offering long-lasting swimwear. In this study, the aging conditions include the exposure of the polymers to water and solar radiation. The aim of the study was to determine the influence of aging on the change in material properties: mass per unit area, thickness, tensile properties, and moisture management.

## 2. Materials and Methods

### 2.1. Materials

For this study, a range of knitted materials were selected consisting of a blend of polymer (polyamide/polyester) and elastane in varying ratios. These specially selected materials are intended for the manufacture of swimwear and are known for their durability, chlorine resistance, multi-directional elasticity for improved comfort, and moderate to strong compression for optimal muscle support. In view of their properties, the 9 materials selected are a representative group of materials available on the market for the intended purpose, in this case, the manufacture of swimwear. Table 1 gives an overview of the materials with IDs in relation to the yarn and knitted fabric parameters.

### 2.2. Material Aging

The protocol for accelerated aging of swimwear materials was established by the authors of this study and used to simulate real wear and tear conditions that occur during sports training in swimming pools. For the definition of the protocol, it was important to gain an insight into the usual training practices, especially the duration of the training, because at different levels of swimming, the intensity of use of a swimsuit increases. A recreational swimmer will perhaps swim 1 to 2 h once or twice a week. But junior cadets (10–12 years old) will train at least 6 h a week, cadets (11–14 years old) 12 h a week, younger juniors (13–16 years old) and juniors (15–18 years old) 12 to 24 h a week, and younger seniors (17–20 years) and seniors (19+) 20 to 24 h a week. The defined protocol focused on three main critical factors that influence material aging—UV radiation, chlorine, and duration of exposure.

To simulate the conditions, the swimwear materials were immersed outdoors in chlorinated swimming pool water. The swimming pool water was prepared from tap water (pH: 7.2–7.5; iron: <20 μg/L; total coliform bacteria: 0 per 100 mL; free residual chlorine: 0.06–0.26 mg/L; color: <5; hardness as CaCO_3_: 336–400 mg/L; specific conductance: 662–772 μS/cm) with the addition of pool chlorine granules (at first 100 g per 10^3^ of water, afterwards 10 g per 10^3^ of water daily). The aging was conducted during the summer season in a Mediterranean climate zone with an average daily temperature of 24 °C, in the shade or exposed to sun. The total durations of immersion were 200 and 300 h. The basis for setting these immersion durations was the intensity of training for juniors, as described in the previous paragraph. Younger juniors or juniors train 20 h a week which reaches an average of 200 h over 10 weeks, i.e., 300 h over 15 weeks. After each immersion, the samples were subjected to 10 washing cycles in a washing machine using a phosphate-free reference detergent with ECE formulation, without optical brighteners. The washing was conducted at a temperature of 30 °C for 20 min and with a centrifugation speed of 800 rpm. The samples were then air-dried in the shade.

### 2.3. Methods

The materials were tested for a range of physical–mechanical properties before and after aging. These properties included mass per unit area, thickness, tensile properties, and moisture management. The tests were carried out as follows:

- Mass per unit area was determined according to method 5 described in [38]. For this purpose, a circular sample with a diameter of 100 mm was weighed on an analytic balance with an accuracy of ± 0.001 g.
- The thickness was tested using the DM-2000 thickness tester (Wolf Messtechnik GmbH, Freiberg, Germany) and in accordance with the standard [39]. For the test, a pressure of 1 kPa was applied to the surface of the test specimen.
- The tensile tests were carried out using the Statimat M tensile tester (Textechno, Mönchengladbach, Germany) in accordance with the standard [40]. The specimen size was 200 × 50 mm and they were cut both in the direction of the courses and in the direction of the wales. A constant tensile force and a test speed of 100 mm/min were maintained for the test. The test was repeated 5 times for each loop direction (wales and courses).
- The moisture management of the materials was tested using the M290 moisture management tester (SDL Atlas, Rock Hill, SC, USA) according to the procedure described in [41]. This experiment focused on wetting time, absorption rate, wetted radius, and overall moisture management capability.

## 3. Results and Discussion

The results include changes in a number of physical–mechanical properties of knitted fabrics due to aging:

- Mass per unit area;
- Thickness;
- Breaking force in the direction of wales (i.e., vertical direction) and in the direction of courses (i.e., horizontal direction);
- Breaking elongation in the direction of wales and courses;
- Moisture management—precisely, the wetting time, wetted radius, and overall moisture management capacity.

All fabric properties were measured before aging, after 200 h of soaking in chlorinated water (designated 200 h Cl), after 200 h of soaking in chlorinated water under the sun (designated 200 h Cl + sun), after 300 h of soaking in chlorinated water (designated 300 h Cl), and after 300 h of soaking in chlorinated water under the sun (designated 300 h Cl + sun).

### 3.1. Changes in Mass per Unit Area After Aging

**Figure 1:**
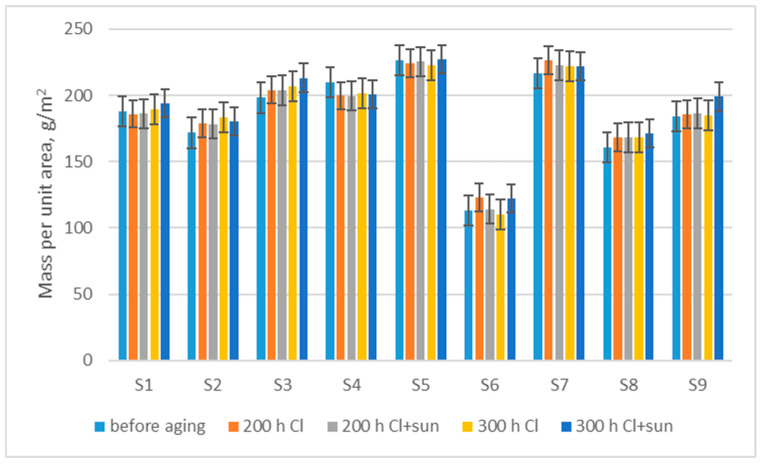
Changes in mass per unit area after aging.

Changes in mass after aging (Figure 1) indicate a tendency for a slight increase in mass, from an average of 1.0% (for exposure 200 h Cl + sun) to an average of 3.7% (for exposure 300 h Cl + sun), as a result of a slight shrinkage of the material after exposure to different aging conditions. If we compare the aging with and without exposure to the sun using the *t*-test, with a significance level of less than *p* < 0.05 (*p* = 0.0250), we can conclude that exposure to the sun’s rays statistically significantly changed the swimwear material after exposure for 300 h, while a statistically significant difference did not occur after 200 h of exposure (*p* = 0.1313).

An estimate of the expected values of mass per unit area, after 200 h of aging in chlorinated water (y), with a certain value of mass per unit area before aging (x), was produced via regression analysis (1).

y = 0.9005x + 21.477; R^2^ = 0.9669 (1)

### 3.2. Changes in Thickness After Aging

**Figure 2:**
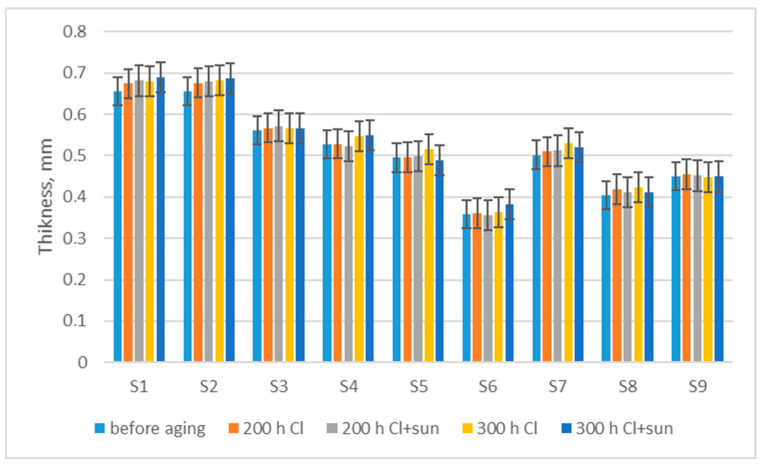
Changes in thickness after aging.

Changes in thickness after aging (Figure 2) show a tendency for a slight increase in thickness, from an average of 1.7% (for exposure 200 h Cl) to an average of 3.2% (for exposure 300 h Cl), which implies a change in the shape of the knitted loop after exposure to different aging conditions. Confirmation of the change in the shapes of the yarn and loops, with filaments that were no longer parallel in the yarn and disorderly shapes of the loops, was also found via microscopic analysis (as shown below). If we compare aging with and without exposure to the sun, the *t*-test shows that the sun did not statistically significantly change the swimwear material thickness even after exposure for 200 or 300 h, while a statistically significant difference occurred due to the exposure time in chlorinated water (200 h, *p* = 0.0044; 300 h, *p* = 0.0008). A previous study [14] shows the influence of the sun on the loss of polymer properties, but it was a matter of longer exposure to the sun.

An estimate of the expected values of thickness, after 200 h of aging in a chlorinated water (y), with a certain value of thickness before aging (x), was produced via regression analysis (2).

y = 1.0427x − 0.0131; R^2^ = 0.9965 (2)

### 3.3. Changes in Breaking Force After Aging

**Figure 3:**
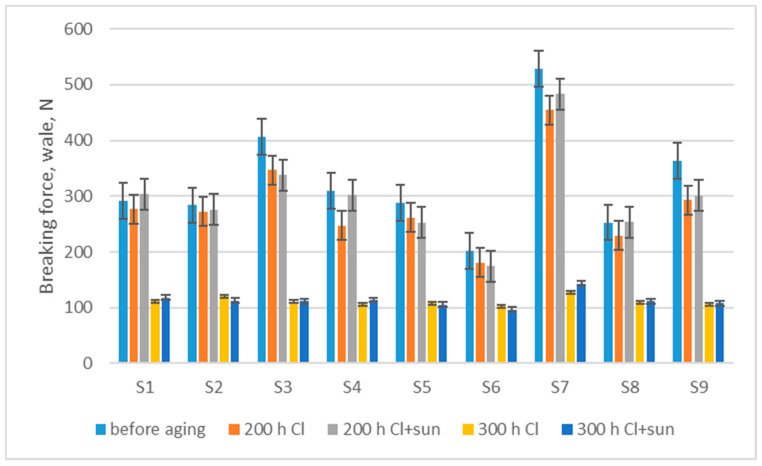
Changes in breaking force measured in the direction of wale, after aging.

After 200 h of aging, all swimwear materials showed a drop in breaking force (12.4% for 200 h Cl, and 8.2% for 200 h Cl + sun). After 300 h of aging, the drop in breaking force was pronounced as a result of fiber and yarn damage (65.7% for 300 h Cl 65.1% for 300 h Cl + sun) (Figure 3). Again, exposure to the sun did not statistically significantly change the material breaking force, while a statistically significant difference occurred due to the exposure time in chlorinated water (200 h, *p* = 0.0007; 300 h, *p* = 0.00005).

**Figure 4:**
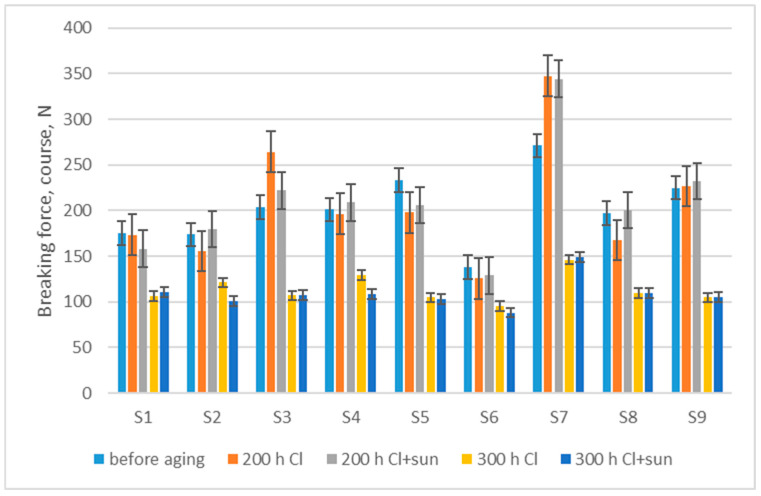
Modifications in breaking force measured in the direction of course, after aging.

After 200 h of aging, the results showed a minimal drop in breaking force in the course direction (200 h Cl—2.0%; 200 h Cl + sun—3.4%). Moreover, with some materials, the breaking force increased, we can assume due to the previously recorded increase in the mass per unit area and thickness (shrinkage) of the swimwear material after aging. After 300 h of aging, the drop in breaking force was clearly visible (300 h Cl—43.7%; 300 h Cl + sun—46.0%) (Figure 4). After 200 h, neither the sun nor the chlorinated water statistically significantly changed the material breaking force (*p* > 0.05), while a statistically significant difference occurred due to the exposure time in chlorinated water after 300 h (*p* = 0.000015). These results are in accordance with previous research on the influence of water or chlorinated water on the properties of polyamide [9,12] or polyester [11,28].

If a correlation is observed of breaking forces in the wale and in the course directions with material properties before aging, there is an extremely high dependence of breaking force after aging on breaking force before aging. Furthermore, there is a medium-strength correlation with the mass per unit area, and there is no correlation with the thickness of the material (Table 2). An estimate of the expected values of the breaking force in the wale direction (3) and course direction (4) after 200 h of aging in chlorinated water (y), with a certain value of breaking force before aging (x), was produced via regression analysis.

y = 0.7933x + 26.994; R^2^ = 0.9587 (3)

y = 1.4892x − 94.733; R^2^ = 0.7414 (4)

**Figure 5:**
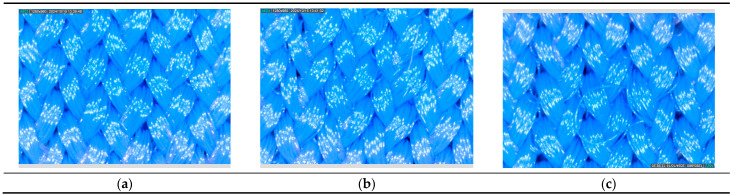
Aging of S8 fabric after 200 and after 300 h in chlorinated water. (**a**) S8 fabric before aging—orderly structure of loops, parallel filaments in the yarn. (**b**) S8 200 h Cl—breaking of few filaments. (**c**) S8 300 h Cl—filaments pulled from the yarn, a disorderly structure of knitted loops.

Changes in the breaking force can furthermore be explained by analyzing changes in the fabric structure. Analysis of the textile structure before and after aging by microscopy with a magnification of 200 times showed fiber and yarn damage, gradual breaking of individual filaments, and a more disorderly structure of the yarn and knitted loops, where the filaments were pulled out of the structure, especially after 300 h of aging (Figure 5).

### 3.4. Changes in Breaking Elongation After Aging

**Figure 6:**
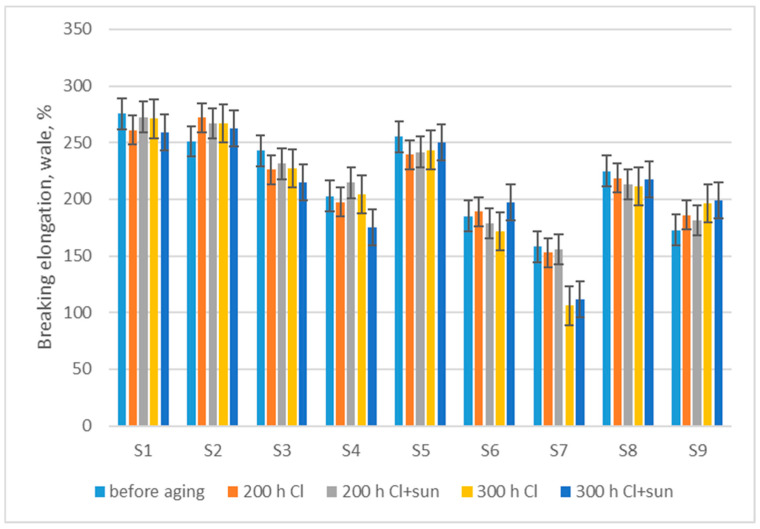
Changes in breaking elongation measured in the direction of wale, after aging.

The measurement of breaking elongation in the direction of wales after aging showed a trend of a small average decrease in breaking elongation, by 1.2% after 200 h Cl, 0.6% after 200 h Cl + sun aging, 3.5% after 300 h Cl, and 4.1% after 300 h Cl + sun aging (Figure 6). Nevertheless, the *t*-test did not show a statistically significant dependence either on aging in chlorinated water or on the sun (*p* > 0.05).

**Figure 7:**
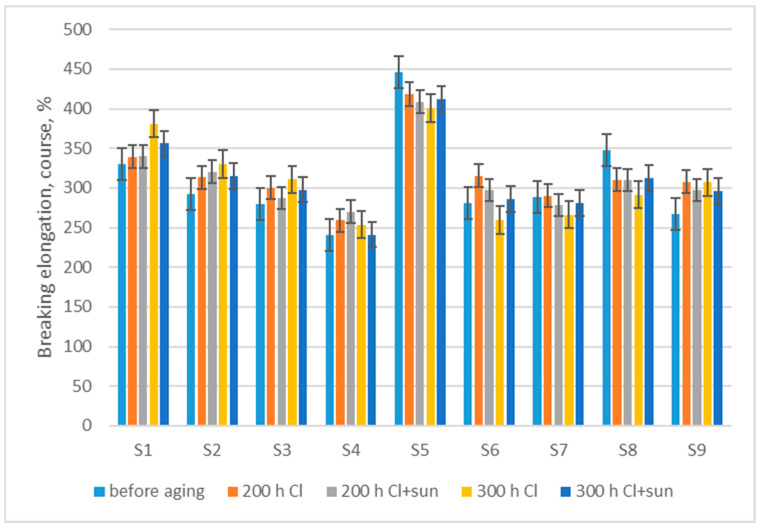
Changes in breaking elongation measured in the direction of course, after aging.

The measurement of breaking elongation in the direction of courses after aging showed a trend of a small average increase in breaking elongation, by 2.9% after 200 h Cl, 1.3% after 200 h Cl + sun aging, 1.0% after 300 h Cl, and 0.9% after 300 h Cl + sun aging (Figure 7). Similar to breaking elongation in the wale direction, the *t*-test did not show a statistically significant dependence of the increase in breaking elongation either on aging in chlorinated water or on the sun (*p* > 0.05).

The correlation of breaking elongation in the wale and in the course directions with material properties before aging showed an extremely high dependence on breaking elongation before aging. There is a medium-strength correlation with thickness, but only in the wale direction, and there is no correlation with mass per unit area of the material (Table 3).

An estimate of the expected values of breaking elongation in the wale direction (5) and course direction (6) after 200 h of aging in chlorinated water (y), with a certain value of breaking elongation before aging (x), was produced via regression analysis.

y = 0.8872x + 21.851; R^2^ = 0.8971 (5)

y = 0.6637x + 112.52: R^2^ = 0.8538 (6)

### 3.5. Changes in Moisture Management

**Figure 8:**
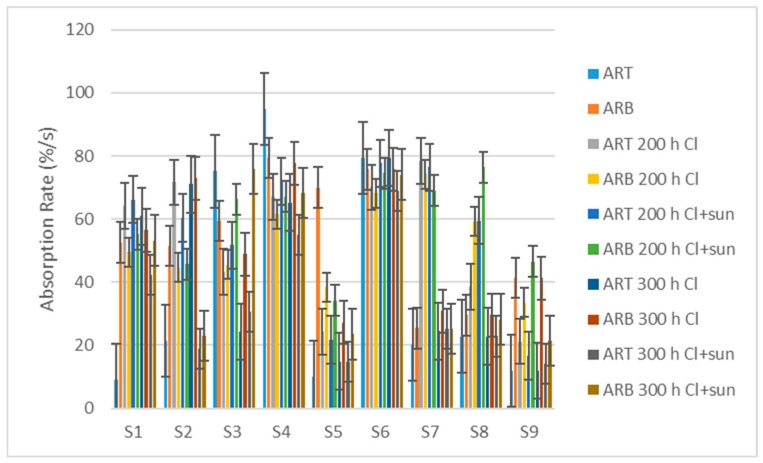
The absorption rate of fabrics.

The values of the absorption rate top (ARt) and absorption rate bottom (ARb) are the average speed of liquid absorption of the tested swimwear material in percentage per second (%/s). Most swimming materials show a low initial absorption rate, which increases as the material ages for 200 h, and then decreases again due to 300 h of aging (Figure 8). The initial low absorption rate is probably due to the water repellent treatment, which wears off, resulting in a higher absorption rate. Further aging impairs the absorption rate of the materials.

**Figure 9:**
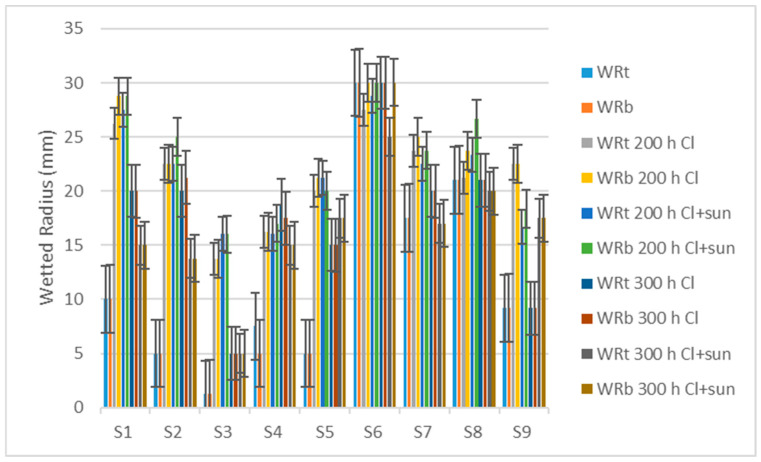
The wetted radius of fabrics.

The wetted radius top (WRt) and bottom (WRb) are the greatest radii of water spreading measured on the top and bottom surfaces of a fabric. Most swimwear materials show less water spread before aging, probably because of the water-repellent treatment (Figure 9). The radius increases after 200 h and decreases again due to 300 h of aging, similar to the absorption rate.

Overall liquid moisture management capability (OMMC) is an index of the overall capability of a fabric to transport liquid moisture. For most of the tested materials, the OMMC index decreases with aging (S1, S2, S4, S7, S9), while a few materials have a similar OMMC index throughout aging (S3, S5, S6, S8) (Table 4). On average, the OMMC value decreases with each aging stage (before aging: 0.4888; 200 h Cl: 0.3457; 200 h Cl + sun: 0.3393; 300 h Cl: 0.3838; 300 h Cl + sun: 0.3253).

## 4. Conclusions

The findings of this study underscore the critical need for advancements in the materials used for swimwear to ensure their greater durability and performance. We have shown that the changes in the properties of polymer materials used in the manufacture of swimwear after 200 h, and especially after 300 h, of exposure to chlorinated water.

1. The mass per unit area increases by up to 3.7% after exposure for 300 h as a result of a slight shrinkage in the material.
2. The thickness of the fabric increases by up to 3.2% after exposure for 300 h because of a change in shape of the knitted loop in swimwear material.
3. The breaking force of swimwear material decreases by up to 12.4% after 200 h of exposure to chlorinated water, and up to 65.7% after 300 h of exposure as a result of fiber and yarn damage. Analysis of the fabric’s structure shows the breaking of individual filaments, and a disorderly structure of the yarn and knitted loops after 300 h of aging. There is an extremely high dependence of breaking force after aging on breaking force before aging and a medium-strength correlation with the mass per unit area.
4. The breaking elongation shows a small decrease in elasticity in the wale direction (up to 4.1%), and a small increase in elasticity in the course direction (up to 2.9%).
5. Changes in moisture management show an initial low absorption rate and less water spread before aging due to the water-repellent treatment, which wears off, resulting in a higher absorption rate and increased water spread after 200 h. Further aging impairs the absorption rate and water spread of the materials.

The presented study highlights critical areas where current swimwear materials fall short, particularly under extended exposure to chlorinated water. For future use, it is essential for producers to innovate and develop materials that can maintain their properties over longer periods. Fabrics of a higher mass, stronger fibers in the yarns, and water repellent treatment that wears off only after a prolonged period will not only improve the durability and performance of swimwear but also enhance the overall user experience.

The goal of this study was to use materials that exist on the market and age them in conditions as similar as possible to real ones in the pool. However, the use of these materials limited this study, and the future scope will be to design yarns and fabrics for swimming from the same polymer but of different masses, and then age them in the same conditions.

## Figures and Tables

**Table 1:** Yarn and knitted fabric parameters.

Fabric ID	Yarn Parameters				Knitted Fabric Parameters	
	Type of Polymer	Yarn Ratio in a Fabric, %		Polymer Fineness, dtex	Mass per Unit Area, g/m^2^	Thickness, mm
		Basic Polymer	Elastane			
S1	polyamide	80	20	110	187.84	0.655
S2	polyamide	80	20	62	171.75	0.655
S3	polyamide	78	22	150	198.13	0.561
S4	polyester	78	22	150	209.59	0.527
S5	polyamide	59	41	44	226.46	0.495
S6	polyamide	73	27	33	113.11	0.358
S7	polyamide	80	20	88	216.52	0.502
S8	polyamide	72	28	44	160.97	0.404
S9	polyamide	71	29	58	184.1	0.450

**Table 2:** Correlation of breaking force after aging and material properties.

	Mass per Unit Area	Thickness	Breaking Force (Before Aging)
F_b_, wale, 200 h Cl	0.61762	0.30903	0.97912
F_b_, wale, 200 h Cl + sun	0.64755	0.32674	0.96057
F_b_, wale, 300 h Cl	0.36220	0.45030	0.67725
F_b_, wale, 300 h Cl + sun	0.53446	0.33150	0.82216
F_b_, course, 200 h Cl	0.65100	0.08850	0.86102
F_b_, course, 200 h Cl + sun	0.63384	−0.00748	0.92321
F_b_, course, 300 h Cl	0.52391	0.27796	0.60326
F_b_, course, 300 h Cl + sun	0.57692	0.15572	0.79535

**Table 3:** Correlation of breaking elongation after aging and material properties.

	Mass per Unit Area	Thickness	Breaking Elongation (Before Aging)
E_b_, wale, 200 h Cl	−0.00385	0.66015	0.94713
E_b_, wale, 200 h Cl + sun	0.14361	0.74594	0.96400
E_b_, wale, 300 h Cl	0.02378	0.59284	0.91288
E_b_, wale, 300 h Cl + sun	−0.15546	0.42292	0.87356
E_b_, course, 200 h Cl	0.17760	0.03078	0.92402
E_b_, course, 200 h Cl + sun	0.22865	0.15430	0.92604
E_b_, course, 300 h Cl	0.35174	0.49723	0.75551
E_b_ course, 300 h Cl + sun	0.23913	0.21035	0.92373

**Table 4:** Overall liquid moisture management capability (OMMC).

			OMMC		
	Before Aging	200 h Cl	200 h Cl + sun	300 h Cl	300 h Cl + sun
S1	0.5861	0.3348	0.3532	0.2813	0.1859
S2	0.6108	0.2048	0.1891	0.2679	0.1753
S3	0.6371	0.5775	0.2316	0.6078	0.6832
S4	0.6919	0.255	0.3293	0.3521	0.2854
S5	0.0244	0.2607	0.2866	0.3578	0.2416
S6	0.4329	0.4000	0.4292	0.4329	0.3969
S7	0.3930	0.3459	0.3250	0.2510	0.1270
S8	0.3468	0.351	0.3759	0.3468	0.3630

## Data Availability

The original contributions presented in this study are included in the article, and further inquiries can be directed to the corresponding author.
